# A Method for Electroporation of Cre Recombinase Protein into Intact *Nicotiana tabacum* Cells

**DOI:** 10.3390/plants12081631

**Published:** 2023-04-12

**Authors:** Yuichi Furuhata, Emiko Egi, Tomi Murakami, Yoshio Kato

**Affiliations:** Biomedical Research Institute, National Institute of Advanced Industrial Science and Technology (AIST), 1-1-1 Higashi, Tsukuba 305-8566, Japan; y-furuhata@aist.go.jp (Y.F.);

**Keywords:** genome engineering, protein delivery, electroporation, Cre recombinase

## Abstract

The Cre/*lox* recombination system has become a powerful technology for gene function analysis in a broad spectrum of cell types and organisms. In our previous report, Cre protein had been successfully delivered into intact *Arabidopsis* thaliana cells using electroporation. To expand the feasibility of the method of protein electroporation to other plant cells, here we attempt the protein electroporation into tobacco-derived BY-2 cells, one of the most frequently used plant cell lines for industrial production. In this study, we successfully deliver Cre protein into BY-2 cells with intact cell walls by electroporation with low toxicity. Targeted *lox*P sequences in the BY-2 genome are recombined significantly. These results provide useful information for genome engineering in diverse plant cells possessing various types of cell walls.

## 1. Introduction

Site-specific DNA recombinases have empowered researchers to manipulate various genes across a broad spectrum of cell types and organisms. In particular, the Cre/*lox* recombination system has become a powerful technology for gene function analysis. In many studies, Cre-mediated recombination has been used to conditionally mutate genes in diverse species [1,2]. Cre recombinase specifically recognizes a 34-bp sequence designated as *lox* (the ‘locus of crossover’) and catalyzes DNA strand exchange between these two sites. Depending on the relative orientation of the *lox* sites to each other, recombination reactions can result in deletions, inversions, insertions, or translocations of chromosomal DNA with high fidelity [2]. Moreover, molecular engineering of Cre-*lox* system allows us to use multiple or orthogonal combinations of recombinase and the recognition sequence different from the original pair to facilitate highly advanced genome engineering such as Tre-*lox*LTR for removal of HIV [3] or Brainbow imaging for neurons [4].

Despite the usefulness, the effectiveness of the Cre/*lox* system depends largely on the availability of a method for delivery of Cre recombinase into cells. In plants, agrobacterium-based gene transfer (AMGT) has been widely used to introduce foreign DNA into plant cells and is the system of choice for genetic transformation of model plants including *Arabidopsis* and *Nicotiana*. However, owing to the biological nature of agrobacterium, transgenes derived from the AMGT vector are randomly integrated into the host genome. This might result in the disruption of host gene expression, since an AMGT-based Cre/*lox* system requires the introduction of a Cre gene in addition to the target *lox* gene. Multiple gene introduction also requires more drug-resistant genes hat their repertoire is limited.

To overcome the limitation of these DNA-based deliveries, protein-based delivery has been attracting attention for biotechnology applications. Previous work in our laboratory involved the introduction of Cre protein into *A. thaliana* cells using electroporation, where we demonstrated the removal of a specific gene fragment in the plant genome [5,6]. Using a suitable buffer, we succeeded in delivering Cre proteins into *Arabidopsis* cells with low cytotoxicity and a high efficiency of over 80%. To evaluate further the reported methodology applicability to other plant cells, here we attempted the protein electroporation into tobacco-derived BY-2 cells. BY-2 cells have become one of the most frequently used plant cell lines for protein production, including for industrial and drug applications, because of their high proliferative nature and scalability [7]. In this study, we successfully delivered Cre protein into BY-2 cells with the intact cell wall by electroporation. The optimal voltage field strength and duration at poring pulse for BY-2 are lower than that for *A. thaliana* cells, reflecting that these cell types have different physical properties on their cell membranes and cell walls.

## 2. Results

**Figure 1:**
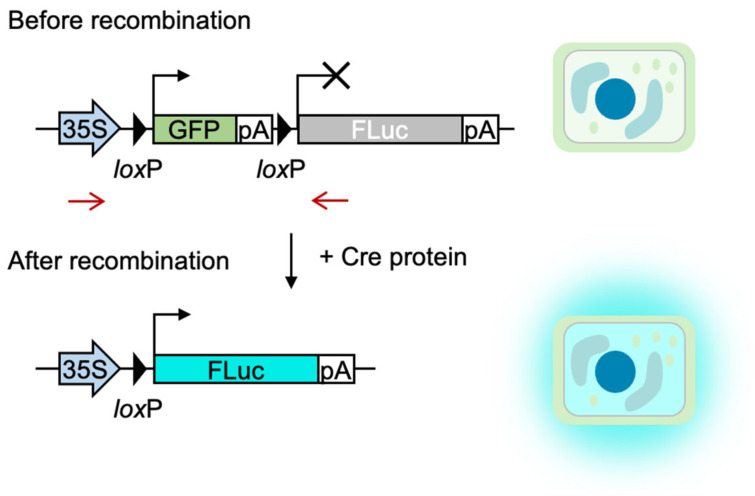
Schematic of Cre reporter design. GFP, pA, and FLuc indicate green fluorescent protein, terminator polyadenylation signal, and firefly luciferase, respectively. Before recombination, the reporter gene is transcribed to synthesize GFP (black arrow) but not FLuc (X). Cre protein catalyzes the recombination between the two *lox*P sites flanking the GFP coding sequence, resulting in FLuc expression that exhibits luminescence upon addition of D-luciferin. The red arrows are the primer-binding sites for genomic PCR.

We first established a reporter cell line (BY-2-xGxFL) to evaluate the delivery efficiency of Cre protein into cells to induce genomic DNA recombination. The reporter cell harbors the gene cassette encoding green fluorescent protein (GFP), followed by a transcription termination signal and a sequence encoding Firefly luciferase (FLuc). We integrated a binary vector T-DNA from pCambiaN-xGxFL into BY-2 with AMGT and selected a hygromycin-resistant clone. The reporter cells originally express GFP under the control of 35S promoter and do not express FLuc. As the result of Cre-induced recombination of two *lox*P sites, which locates up and down stream of GFP, BY-2-xGxFL cells come to express FLuc and, thus, quantified as luminescence (Figure 1). This method is much more reliable and quantitative as the proof method for intracellular delivery compared with the microscopic observation of fluorescently labeled protein, which may experience background fluorescence [8]. A luminescence signal is acquired only when Cre proteins get internalized into cells and mediate the recombination reaction at the host cell genome.

**Figure 2:**
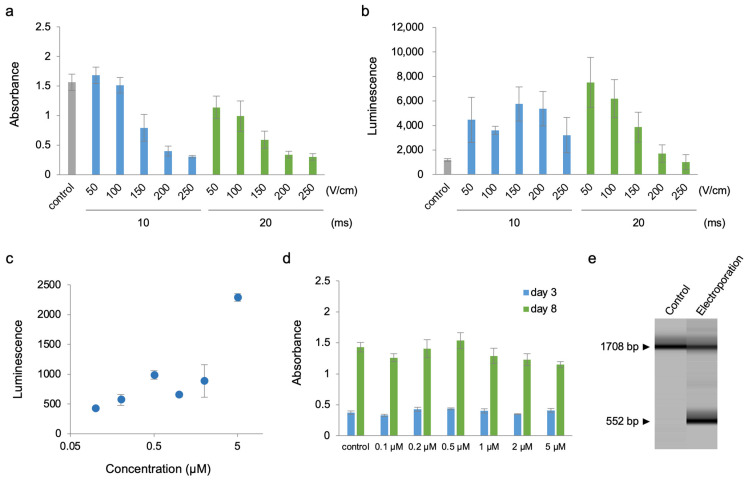
Electroporation-mediated Cre protein delivery into BY-2 cells. (**a**) Effect of poring pulse conditions on the viability of BY-2-xGxFL cells. CCK8 assay was performed 2 d after electroporation in electroporated BY-2-xGxFL cells using different poring pulse conditions: field strength (50, 100, 150, 200, or 250 V/cm), duration (10 or 20 ms) by measuring the absorbance at 450 nm. Control indicates untreated BY-2-xGxFL cells. Values shown are the mean  ±  SE of *n*  =  3. (**b**) Effect of poring pulse conditions on electroporation efficiency. Firefly luciferase activity was determined by the luminescence measurement of catalyzed D-luciferin in electroporated BY-2-xGxFL cells using different poring pulse conditions: field strength (50, 100, 150, 200, or 250 V/cm), duration (10 or 20 ms). In all cases, 5 μM Cre protein dissolved in Opti-MEMI was used for electroporation. Control indicates untreated BY-2-xGxFL cells. Values shown are the mean  ±  SE of *n*  =  3. (**c**) Effect of Cre protein concentration on on electroporation efficiency. Firefly luciferase activity as determined by the luminescence measurement of catalyzed D-luciferin in electroporated BY-2-xGxFL cells with different concentrations of Cre protein dissolved in Opti-MEMI; 0.1, 0.2, 0.5, 1.0, 2.0, or 5.0 μM. Values shown are the mean  ±  SE of *n*  =  3. (**d**) Viability of electroporated BY-2-xGxFL cells with different concentrations of Cre protein. CCK8 assay was performed 2 d after electroporation by measuring the absorbance at 450 nm. Control indicates untreated BY-2-xGxFL cells. Values shown are the mean  ±  SE of *n*  =  3. (**e**) Microchip electrophoresis analysis of genomic DNA PCR products from BY-2-xGxFL cells. The representative image in three independent experiments is shown. The 1708 bp and 552 bp fragments represent the reporter gene cassette before and after Cre-mediated recombination, respectively.

To deliver Cre protein into BY-2 cells with an intact cell wall, the purified Cre protein was subjected to the reporter cell line BY-2-xGxFL with electroporation. Cre proteins were genetically fused with an N-terminal nuclear localization signal (NLS) and hexa-histidine tag. In our previous reports, we observed efficient delivery for *A. thaliana* T87 cells at the condition with 375 V/cm of field strength, 10 msec of poring pulse, and 5 times of multiple pulses in the presence of an Opti-MEMI buffer using an NEPA21 TYPE II apparatus [5]. We initially attempted the same condition for tobacco BY-2 cells as T87 cells; however, the BY-2 cells were largely damaged. Then we re-examined the electroporation conditions for BY-2 cells with a milder field strength of the poring pulse. The cytotoxicity caused by the poring pulse increased in response to increases in field strength when BY-2 cells were subjected to electroporation and was severe even at 200 V/cm of the field strength (Figure 2a). We obtained the optimal condition at 50 V/cm of the field strength and 20 ms duration of the poring pulse (Figure 2b). At this condition, reporter cells after treatment of protein electroporation exhibited less cytotoxicity and the highest luminescence among tested.

Next, we assessed the effect of Cre protein concentration on the electroporation efficiency. Cells were electroporated with 0.01–5 μM of Cre protein using the optimal electroporation program. As a result, Cre protein delivery increased luminescence concomitantly with the concentration of Cre protein (Figure 2c). Higher concentration of Cre protein administration did not largely affect the cytotoxicity over 3–8 days after electroporation (Figure 2d). These results reveal that protein concentration was an important factor in the efficacy of protein electroporation into cells.

To further validate the Cre protein delivery, we analyzed genomic DNA from the reporter cells with or without protein electroporation. A pair of PCR primers was designed to bind the regions of both the 35S promoter and the gene for FLuc (Figure 1; red arrows). The resultant PCR products, estimated as 1708 bp for the non-treatment control and 552 bp for recombined sequence, were quantified as electrogram by capillary electrophoresis. As shown in Figure 2e, electroporated cells exhibited a significant ratio on the PCR product, indicating that the electroporation at the optimal condition delivered the Cre protein into cells and induced the recombination at the targeted DNA sequence.

## 3. Discussion

Using the electroporation method, we introduced the Cre protein into tobacco cells, and observed the activity of the introduced protein through the genome recombination event. The characteristics of the protein of interest, concentration, or electroporation conditions can greatly affect the efficiency of delivery depending on the types of cargo proteins and target cells. In a previous report, Cedeño et al. attempted the delivery of ERD proteins into tobacco cells to analyze the in-cell NMR through the cell wall using electroporation [9]. Fluorescently labeled ERD proteins (250 uM, 20–30 kDa, and 5.1–5.4 of pI) were electroporated with a 750 V/cm and 20 ms poring pulse condition. Under our experimental condition, BY-2 cells were severely damaged with the poring pulse over 250 V/cm and 20 ms, although the conditions are not identical because of the different electroporation devices. Researchers may need to consider the copy number of the target sequences and to screen optimal parameters according to the specific cell types.

Unlike the introduction of DNA, the introduction of proteins into plant cells with intact cell walls brings various advantages. In particular, DNA-free genome modification or editing technology is expected to play an important role in plant engineering. For example, long-term expression of artificial nucleases such as CRISPR-Cas9 or TALENs induces harmful actions for the host genome by off-target effects. The Cre-*lox*P system as a method of removing unnecessary genes is an appropriate option for BY-2 cells, which are promising in the production of drug substances or biopharmaceuticals, since null segregations by crossing are not applicable. Thus, it may be possible to prevent such side effects simply by introducing the artificial nuclease proteins or by removing the gene cassette with the Cre protein. Based on our results, we anticipate that BY-2 cells will be more industrially valuable through the gene network engineering by direct protein delivery.

## 4. Methods

### 4.1. Plasmid Construct Preparation

To construct pCambiaN-xGxFL, used for generation of BY-2 reporter cells, a DNA fragment encoding Firefly luciferase was amplified with the PCR primer set (5′- TACGAAGTTATCTAGACCATGGAAGATGCCAAAAACATT-3′ and 5′- GTCACCAATTCACACGTGTTACACGGCGATCTTGCCGCC-3′) and integrated into pCambiaN-xGxGUS^5^ at XbaI and PmlI sites to replace the GUS gene. The complete sequence of pCambiaN-xGxFL is shown in Figure S1.

### 4.2. Preparation of Cre Protein

HNCre protein was expressed using *Escherichia coli* strain BL21(DE3; Nippon Gene, Tokyo, Japan) carrying pET-HNCre, encoding a hexa-histidine tag and nuclear localization signal at the N-terminus followed by Cre recombinase. This Cre gene carries the A207T mutation, while its recombination activity is comparable to that of the wild type [5]. The overnight starter culture of *E. coli* BL21(DE3) cells were diluted 100-fold into LB broth (Lennox; Merck, Darmstadt, Germany) medium supplemented with 10 µg/mL kanamycin and incubated at 37 °C until A_600_ reached 0.6. The cells were then incubated with 0.1 mM isopropyl β-D-1-thiogalactopyranoside for another 2.5 h at 37 °C to induce protein expression and then harvested. Cells were then lysed with Digital Sonifier 250D Advanced (Branson, CT, USA) and applied to an Ni-NTA column with elution buffer (50 mM Tris-HCl, 500 mM NaCl, 10% glycerol, and 500 mM imidazole, pH 8.0). HNCre protein was further purified using a gel filtration column (HiPrep 16/60 Sephacryl S-200 HR; GE healthcare, Chicago, IL, USA) with Buffer A (20 mM HEPES, 500 mM NaCl, 10% glycerol, and 1 mM dithiothreitol, pH 7.4). The purified HNCre protein was flash-frozen in liquid N_2_ and stored at −80 °C until further use.

### 4.3. BY-2 Cell Line and Agrobacterium Tumefaciens (Rhizobium radiobacter) Culture

The *Nicotiana tabacum* BY-2 cell line was obtained from the RIKEN Bio Resource Center (Ibaraki, Japan). BY-2 cells were cultured in a liquid LS culture medium at 22 °C while shaking (120 rpm) in the dark. The LS culture medium consists of the following ingredients: 30 g/L sucrose, 200 mg/L KH_2_PO_4_, 1× Murashige and Skoog plant salt mixture (FUJIFILM Wako Pure Chemical, Osaka, Japan), 40 g/L myo-inositol and 400 mg/L thiamine hydrochloride, and 904 nM 2,4-dichlorophenoxyacetic acid, with pH 5.7 adjusted with KOH solution. Cells were maintained using weekly 30- to 60-fold dilutions.

### 4.4. Agrobacterium-Mediated Transformation of BY-2 Cells

BY-2-xGxFL cells, the BY-2 cell line carrying Cre-responsive *Firefly* luciferase reporter cassette, were generated using AMGT, as reported previously, with minor modifications (An, Plant. Physiol, 1985). In brief, BY-2 cells were mixed with *Agrobacterium* cells harboring pCambiaN-xGxFL in the presence of 100 µM acetosyringone. Two days post-infection, the cells were washed using culture medium supplemented with 200 µg/mL cefotaxime to eliminate EHA105 and were transferred onto LS agar (LS medium with 0.7% agar) with 200 µg/mL cefotaxime and 40 µg/mL hygromycin and cultured at 28 °C in the dark for 2 weeks. A single clone was then picked and cultured in liquid LS medium containing 20 µg/mL hygromycin.

### 4.5. Electroporation

BY-2-xGxFL cells were collected in a tube and washed once with Opti-MEM I (Thermo Fisher Scientific, Waltham, MA, USA). Cells with 20 µL of PCV were suspended in 200 µL of Opti-MEM I with HNCre protein on ice. HNCre protein concentrations were 5 µM to evaluate the poring pulse voltage and length effect, were 0.1, 0.2, 0.5, 1, 2, or 5 µM to evaluate the protein concentration effect, and were 2 µM to evaluate the recombination efficiency with genomic PCR. Cells were then transferred into a 4 mm wide cuvette (Nepa Gene, Chiba, Japan). To evaluate the effect of voltage and pulse length of the poring pulse, the poring pulse program was 50, 100, 150, 200, or 250 V/cm (20, 40, 60, 80, or 100 V setting/0.4 cm width), 10 or 20 ms, 5 times, 10% of decay rate, and a 50 ms interval. In the other experiments, the poring pulse program was 50 V/cm (20 V setting/0.4 cm width), 20 ms, 5 times, 10% of decay rate, and a 50 ms interval. The transfer pulse program was 20 V, 20 ms, 20 times, 10% of decay rate, and a 50 ms interval for all the experiments. Electroporation was performed using the NEPA21 Type Ⅱ (Nepa Gene). Immediately after electroporation, the cells were washed once with LS medium and cultured in 2 mL of LS medium at 22 °C with shaking (120 rpm) in the dark until they were used for further experiments.

### 4.6. Luminescence Quantification in Cells

Two days after electroporation, the volume of each cell culture was reduced to 800 µL by discarding the supernatant, and 80 µL of each cell suspension was transferred to a white 96-well plate (Thermo Fisher Scientific). Subsequently, cells were added to 40 µL of Dual-Glo Luciferase Reagent (Promega, Madison, WI, USA) and shaken with a microplate mixer (NS-4P, ASONE, Osaka, Japan) at 25 °C for 5 min. The plate was then turned around and shaken for another 5 min. After incubation, luminescence was measured using a microplate reader (Infinite 200 Pro; Tecan, Zurich, Switzerland).

### 4.7. Cell Counting Kit-8 (CCK8) Assay

Cell number was analyzed using the Cell Counting Kit-8 (Dojindo Laboratories, Kumamoto, Japan). Two and seven days after electroporation, 100 µL each of the cell suspensions was transferred to a 96-well plate to which 10 µL of CCK8 solution was added. The cells were incubated at 22 °C with shaking (120 rpm) in the dark for 3 h. Absorbance at 450 nm was measured using a microplate reader (Infinite 200 Pro; Tecan) to evaluate the number of cells.

### 4.8. Genomic PCR of Cre-Responsive Reporter Cassette

BY-2 cells 7 d after electroporation were incubated with 40 µL of 250 mM NaOH and 0.1% Tween 20 at 98 °C for 10 min to extract genomic DNA [5]. The samples were then treated with 20 µL of 1 M Tris-HCl (pH 6.5) and centrifuged. Using the extracted genomic DNA, the integrated sequence of Cre-responsive reporter cassette in BY-2 genome was amplified via PCR using a KOD One PCR Master Mix (TOYOBO; Osaka, Japan, 35 cycles each of 98 °C for 10 s, 62 °C for 5 s, and 68 °C for 1 min 30 s) with the primers 5′-TGGAGCACGACACACTTGTCTAC-3′ and 5′-AGCTGCTCGCCGGCGGTCCC-3′. The PCR products were analyzed with a 1 kb Plus DNA Ladder (New England Biolabs, Ipswich, MA, USA) using MultiNA (MCE202, SHIMADZU, Kyoto, Japan).

### 4.9. Quantification and Statistical Analyses

All measurements are presented as mean ± standard error (SE). *p* values in figures were obtained by a Student’s *t*-test. Sample sizes are indicated in the figure legends.

## Data Availability

The data of this study are available from Yoshio Kato upon request.

## Supplementary Materials

The following supporting information can be downloaded at: https://www.mdpi.com/article/10.3390/plants12081631/s1, Figure S1: DNA sequence of the pCambiaN-xGxFL plasmid.
